# An in-depth report of quality control on Kato-Katz and data entry in four clinical trials evaluating the efficacy of albendazole against soil-transmitted helminth infections

**DOI:** 10.1371/journal.pntd.0008625

**Published:** 2020-09-21

**Authors:** Johnny Vlaminck, Piet Cools, Marco Albonico, Shaali Ame, Mio Ayana, Daniel Dana, Jennifer Keiser, Leonardo F. Matoso, Antonio Montresor, Zeleke Mekonnen, Rodrigo Corrêa-Oliveira, Simone A. Pinto, Somphou Sayasone, Jozef Vercruysse, Bruno Levecke

**Affiliations:** 1 Department of Virology, Parasitology and Immunology, Ghent University, Merelbeke, Belgium; 2 Center for Tropical Diseases, Sacro Cuore Don Calabria Hospital, Negrar, Italy; 3 Department of Life Sciences and Systems Biology, University of Turin, Turin, Italy; 4 Laboratory Division, Public Health Laboratory-Ivo de Carneri, Chake Chake, United Republic of Tanzania; 5 Jimma University Institute of Health, Jimma University, Jimma, Ethiopia; 6 Department of Medical Parasitology and Infection Biology, Swiss Tropical and Public Health Institute, Basel, Switzerland; 7 University of Basel, Basel, Switzerland; 8 Laboratory of Molecular and Cellular Immunology, Research Center René Rachou—FIOCRUZ, Belo Horizonte, Brazil; 9 Nursing school, Federal University of Minas Gerais, Minas Gerais, Brazil; 10 Department of Control of Neglected Tropical Diseases, World Health Organization, Geneva, Switzerland; 11 Lao Tropical and Public Health Institute, Ministry of Health, Vientiane, Lao People's Democratic Republic; Imperial College London, Faculty of Medicine, School of Public Health, UNITED KINGDOM

## Abstract

**Background:**

Efforts to control soil-transmitted helminth (STH) infections have intensified over the past decade. Field-survey data on STH prevalence, infection intensity and drug efficacy is necessary to guide the implementation of control programs and should be of the best possible quality.

**Methodology:**

During four clinical trials designed to evaluate the efficacy of albendazole against STHs in Brazil, Ethiopia, Lao PDR and Tanzania, quality control (QC) was performed on the duplicate Kato-Katz thick smears and the data entry. We analyzed datasets following QC on both fecal egg counts (FECs) and data entry, and compared the prevalence of any STH infection and moderate-to-heavy intensity (MHI) infections and the drug efficacy against STH infections.

**Results:**

Across the four study sites, a total of 450 out of 4,830 (9.3%) Kato-Katz thick smears were re-examined. Discrepancies in FECs varied from ~3% (hookworms) to ~6.5% (*Ascaris lumbricoides* and *Trichuris trichiura*). The difference in STH prevalence and prevalence of MHI infections using the datasets with and without QC of the FECs did not exceed 0.3%, except for hookworm infections in Tanzania, where we noted a 2.2 percentage point increase in MHI infections (pre-QC: 1.6% *vs*. post-QC: 3.8%). There was a 100% agreement in the classification of drug efficacy of albendazole against STH between the two datasets.

In total, 201 of the 28,980 (0.65%) data entries that were made to digitize the FECs were different between both data-entry clerks. Nevertheless, the overall prevalence of STH, the prevalence of MHI infections and the classification of drug efficacy remained largely unaffected.

**Conclusion/significance:**

In these trials, where staff was informed that QC would take place, minimal changes in study outcomes were reported following QC on FECs or data entry. Nevertheless, imposing QC did reduce the number of errors. Therefore, application of QC together with proper training of the personnel and the availability of clear standard operating procedures is expected to support higher data quality.

## Author summary

Over the past ten years, there has been a worldwide intensification of large-scale deworming programs aimed at controlling the morbidity of intestinal worms (roundworms, whipworms and hookworms). In order to make well-founded decisions, it is of utmost importance that the program managers have access to high quality data on the prevalence and intensity of these infections, and the therapeutic efficacy of the administered drugs. In the present study, we report the results of the quality control (QC) that was performed on the diagnosis and the data entry. We analyze and discuss how QC affected the study outcomes of four clinical trials designed to assess the therapeutic efficacy of a standard deworming treatment in Brazil, Ethiopia, Lao PDR and Tanzania. Our results indicate that, when staff is aware that QC will be performed, study outcomes were not significantly affected by performing QC on both the diagnosis or the data entry. Nevertheless, it is important to highlight that training, adherence to good standard operating procedures and performing QC does improve the overall quality of the data.

## Introduction

An effective control program for soil-transmitted helminths (STHs; *Ascaris lumbricoides*, *Trichuris trichiura*, *Necator americanus* and *Ancylostoma duodenale*) is steered by data obtained from studies that monitor infection prevalence and intensity or evaluate the therapeutic efficacy of anthelmintic drugs. This is because the output of such studies is compared to WHO reference values and, depending on the results, the control program is adapted [1–3]. Therefore, it is essential that the data collected during such studies is of the highest possible quality. Good-quality data is not only the result of a good diagnostic analysis, but also requires the obtained data to be processed correctly.

To ensure the accuracy of the results obtained during diagnostic microscopy, a rigorous quality control (QC) can be performed [2, 4]. Today, in programmatic settings, the Kato-Katz thick smear is still the most widely applied method to diagnose STH infections. In the WHO’s manual on the assessment of anthelmintic drug efficacy, it is suggested to re-examine a random 10% subset of samples by an expert microscopist for QC purposes [2, 4]. If the expert identifies a difference in fecal egg counts (FECs) of more than 10% and more than four eggs, it is suggested to re-examine the slide and to discuss discrepancies. Yet, no specific guidance is provided on how to select these samples or how to deal with discrepancies. Investigators at the Swiss Tropical and Public Health Institute (Swiss TPH) noted that the QC discrepancy criteria for egg counts presented by WHO [2] were also too strict [4]. As a response, they put forward their own recommendations on how to define and judge discrepancies in FECs between the initial reading and the QC reading of Kato-Katz thick smears [4] (**Table 1**). However, today, rigorous QC is hardly ever enforced or performed [4] and if it is performed, results are only seldomly reported. Consequentially, the impact of QC of the diagnostic analysis on study results is unknown.

**Table 1:** WHO and Swiss TPH guidelines for quality control of fecal egg counts based on Kato-Katz. (Adopted from WHO, 2013 [2] and Speich et al. 2015 [4]).

World Health Organization (WHO)	Swiss Tropical and Public Health Institute (Swiss TPH)
**“If the expert identifies a difference in the egg count per gram of stool** ^**a**^ **of more than 10% and more than four eggs, he or she should re-read the slide with** **the microscopist and discuss the reasons for the discrepancy”.** ^**b**^	Results are considered as inconsistent if: (i) there is a difference in presence/ absence of a specific helminth species, (ii) if differences in egg counts exceed 10 eggs for Kato-Katz thick smears with ≤100 eggs, (III) if differences in egg counts exceed 20% for Kato-Katz thick smears with more than 100 eggs.

^a^ To calculate eggs per gram of stool, the egg counts from a single Kato-Katz thick smear are multiplied by a factor of 24. Therefore, differences in egg counts per gram of stool of less or equally to four eggs are not possible. We assume that the WHO aimed to apply their guideline for egg counts for Kato-Katz thick smears rather than per gram of stool (as it is indicated within a footnote of the WHO document).

^b^ WHO guidelines do not explicitly state how to handle discrepancies.

The application of a rigorous data management system is equally critical in the generation of high-quality datasets [5, 6]. Errors are easily introduced during the data entry process and may potentially result in faulty study results. Though ICH-GCP guidelines do not require a specific data entry process, one technique that has shown to ensure a lower number of data entry errors in computer data sets is the use of a double data entry system. Here, data is entered twice by either the same individual or two separate individuals [7].

In this manuscript, we provide an in-depth report of quality control on Kato-Katz and data entry in four clinical trials evaluating the efficacy of albendazole against STH infections. For this, we analyzed study datasets obtained before and after QC on FECs or data entry and compared the obtained results with regards to STH infection prevalence of any intensity, the prevalence of MHI infections and drug efficacy.

## Methods

### Ethics statement

The data was collected during clinical trials in Brazil, Ethiopia, Lao PDR and Tanzania. [8]. The study protocol for these trials was reviewed and approved by the Institutional Review Board (IRB) of the Faculty of Medicine and Health Sciences of Ghent University, Belgium (Ref. No B670201627755; 2016/0266) and by responsible national ethical committees associated with each trial site (Ethical Review Board of Jimma University, Jimma, Ethiopia: RPGC/547/2016; National Ethics Committee for Health Research (NECHR), Vientiane, Lao PDR: 018/NECHR; Zanzibar Medical Research and Ethics Committee, United Republic of Tanzania: ZAMREC/0002/February/2015 and the IRB from Centro de Pesquisas René Rachou, Belo Horizonte, Brazil: 2.037.205). Parent(s)/guardians of participants signed an informed consent document indicating that they understood the purpose of and procedures required for the study, and that they allowed their child to participate. If the child was ≥5 years, he or she had to orally assent in order to participate. Participants of ≥12 years of age were only included if they signed an informed consent document indicating that they understood the purpose of the study and the procedures required for the study, and were willing to participate. The trial was retrospectively registered on Clinicaltrials.gov (ID: NCT03465488) on March 7, 2018.

### Study design and procedures

The four clinical trials were designed to assess an equivalence in therapeutic efficacy of a single oral dose of 400 mg albendazole against STH infections in school aged children (SAC) measured by a variety of diagnostic methods. At the start of each trial, schools were visited by the local principal investigator and a team of field officers, who explained the planned trial and sampling method to the parents and teachers and the children. At baseline, demographic data was collected of the SAC that consented to join the trial and they were asked to provide a fresh stool sample. Upon delivery of the stool sample they were treated with a single oral dose of 400 mg of albendazole under supervision. All collected stool samples were processed to determine FECs (expressed in eggs per gram of stool (EPG)) for each STH using duplicate Kato-Katz as previously described [8] (**S1 Info**). Fourteen to 21 days after drug administration, a second stool sample was collected from all the children that were found positive for any STH at baseline. Stool samples collected at follow-up were again examined by duplicate Kato-Katz. During duplicate Kato-Katz examination, the number of STH eggs counted in each of the two Kato-Katz slides (slide A and B) were recorded separately (**S2 Info**). To reduce microscopist-associated bias, microscopists were not allowed to examine both Kato-Katz slides of the same sample.

### Quality control on fecal egg counting

QC was performed as put forward by Speich and coworkers [4]. In brief, the selection of which samples to re-examine for QC was predetermined by providing a list of 80 random numbers between 0 and 800 (**S3 Info**). This was done to ensure a 10% re-examination rate and to prevent selection bias. Both Kato-Katz thick smears (A and B) were re-examined by a second reader who was blinded to the initial FECs. The results of the QC were recorded on separate record forms (**S2 Info**) to compare with the original egg count data. The internal recommendations of the Swiss TPH were used as a guide to define and classify discrepant results [4] (see **Table 1**). The results were defined and classified into the following four types of discrepancies: (i) type 1: detection of a false positive or negative result, (ii) type 2: re-examined slide shows a difference >10 eggs when less than 100 eggs were counted during the first reading or (iii) type 3: a difference >20% when 100 eggs or more were counted during the first reading; (iv) finally, there could also be an absence of any type of discrepancy. In the absence of discrepancies between both readings, the original FECs were kept. In case of discrepant results, a third examiner re-examined the slide. In the case of a type 1 discrepancy, the presence or absence of eggs of the STH species in question was verified. In case of type 2 or 3 discrepancies, the FECs were compared to the FECs obtained by the first reader and the reader that performed QC. The FEC value of the reader that was closest to the FEC obtained during the third reading was considered as correct. If the FEC of the QC reading was considered as correct, the original recorded FEC was changed to the FEC value obtained during QC. A detailed standard operating procedure (SOP) on the performance of QC and how we resolved QC issues is provided in the supplementary information (**S3 Info**).

### Quality control on data entry

During the clinical trials, paper case report forms were used to record the results of the Kato-Katz thick smears and the QC (**S2 Info**). Data was digitalized by two independent data entry clerks (either laboratory technicians or other personnel) by copying data into a customized data entry file (DEF) prepared in Microsoft Excel (**S4 Info**). The data fields were clearly defined in the header and were consistent throughout the different sheets. No other modifications to the DEF were made. For the duplicate Kato-Katz results, six entries were made per sample, namely the FECs for *Ascaris lumbricoides*, *Trichuris trichiura* and hookworm on the two thick smear slides. Upon completion of the data entry, the DEFs from the two clerks were compared electronically for agreement using the “Inquire” add-in for Microsoft Excel (**S5 Info**). When differences were detected, the correct values were determined by checking the original paper forms. Differences between the DEFs of both data entry clerks that were the consequence of misspelling or formatting (e.g. cell or text color, different cell format, etc.) were not considered as errors.

### Statistical analysis

#### Quality Control on fecal egg counting

For the QC of the FECs, the proportion of the different types of discrepancies was calculated on a country- and STH-specific basis. The overall STH prevalence and prevalence of moderate-to-heavy intensity (MHI) infections, and the therapeutic efficacy were calculated using the two data files. One containing the original FECs (obtained after first reading) and one with corrected FECs following QC on 10% of the slides. For this, only the duplicate Kato-Katz data collected at baseline was used. Infection intensities were classified into light or MHI according to WHO thresholds, MHI infections being defined when FECs ≥5,000 EPG (*A*. *lumbricoides*), ≥1,000 EPG (*T*. *trichiura*) and ≥2,000 EPG (hookworm) [3]. To evaluate the drug efficacy, the egg reduction rate (ERR) was calculated separately for each helminth species. The ERR was calculated using the population of study participants that were positive for that respective STH during baseline survey and who also provided a stool sample for analysis during the follow-up survey. The ERRs were calculated as follows: ERR (%) = 100 x (1 - (arithmetic mean of post-intervention FECs / arithmetic mean of pre-intervention FECs)). Drug efficacy levels were classified according to WHO reference values for albendazole into satisfactory, doubtful or reduced [2].

#### Quality Control on data entry

The number of discrepancies in the Kato-Katz results (both baseline and follow-up results) entered by the two data entry clerks was counted and expressed as a percentage of the total number of entries made per site. The overall STH prevalence infections of any intensity, prevalence of MHI infections, and the ERR were calculated for each DEF (DEF from person 1, DEF from person 2 and the final DEF with the consolidated and corrected data) and results were compared across the data sets. Analysis were done with data from each study site (Brazil, Ethiopia, Lao PDR and Tanzania). Statistical analyses were conducted in R, Microsoft Excel v16.16.7 or Prism version 6.0. for Mac.

## Results

### Quality control on fecal egg counts

#### Number of re-examinations performed during quality control on fecal egg counts

A total of 2,415 individual stool samples were examined during the studies across the four endemic countries. These included the stool samples screened during baseline and follow-up. Fewer samples (n = 285) were examined in Brazil compared to the other three countries (Ethiopia: 748, Lao PDR: 776 and Tanzania: 606). This difference in sample size was the result of a premature termination of the trial due to unexpected low prevalence of any STH infections in the study area. Across the four study sites, a total of 9.3% of Kato-Katz thick smears (= 450 smears from 225 samples) were re-examined (Brazil: 7.4% (21/285), Ethiopia: 14.4% (108/748), Lao PDR 7.0% (54/772) and Tanzania: 7.0% (42/604)). Ethiopia exceeded the requested number of samples to re-examine as they voluntarily performed QC on more samples.

#### Number of discrepancies detected through quality control on the fecal egg counts

In total, 450 Kato-Katz thick smears were re-examined as part of the QC across the four study sites. This includes 296 slides re-examined during baseline and 154 slides re-examined during follow-up. In these studies, we followed the recommendations put forward by Swiss TPH to evaluate QC and to see whether a third reading of the slide was needed to provide a definite answer. The QC revealed discrepancies in 65 out of the 450 slides (14.4%). The number of slides in which discrepancies were detected was higher at baseline (16.2%) than at follow-up (11.0%).

**Fig 1:**
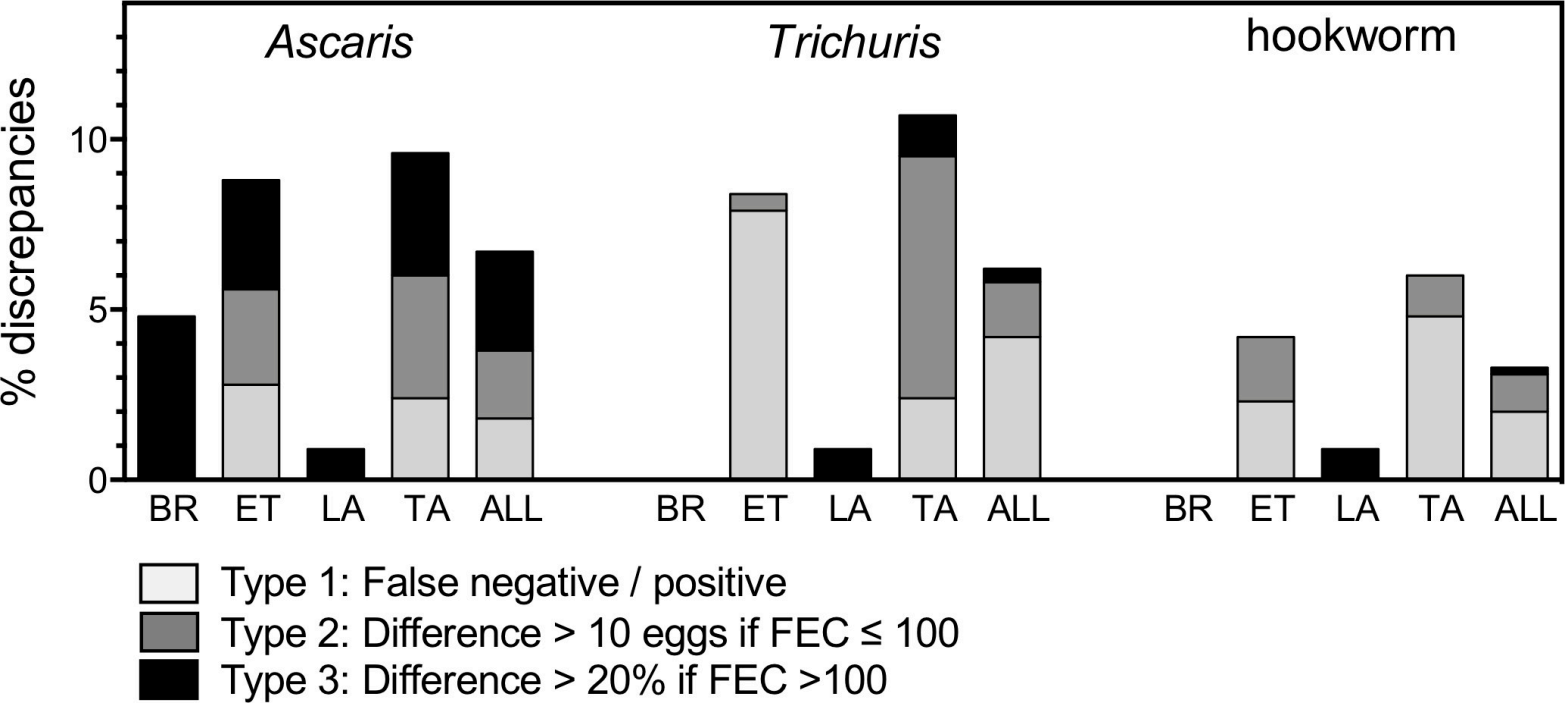
Overview of the percentage and type of discrepancies observed during the quality control on fecal egg counts. The proportion and type of discrepancies detected during quality control of duplicate Kato-Katz thick smears are grouped per helminth species. The type of discrepancy (type 1–3) is indicated by color code and is presented per country (BR: Brazil (n = 42); ET; Ethiopia (n = 216); LA: Lao PDR (n = 108); TA: Tanzania (n = 84)) and as a proportion across the four different studies (ALL: all countries combined; n = 450).

The results of the re-examinations per STH species and per country are presented in **Fig 1.** On average, a total of 6.7% (30/450), 6.2% (28/450) and 3.3% (15/450) discrepant FECs were detected for *Ascaris*, *Trichuris* and hookworm respectively. Tanzania, the site with the highest prevalence of STH infections (99%) and mean STH FECs per sample [9], also showed most discrepancies for all three STHs (*Ascaris*: 9.5%, *Trichuris*: 10.7% and hookworm: 6.0%). In Ethiopia, a similar level of discrepancies (*Ascaris*: 8.8%, *Trichuris*: 8.3% and hookworm: 4.2%) was detected across the three different STH species. In Lao PDR, only one type 3 (difference >20% when FECs >100 eggs) discrepancy was detected for *Trichuris* during re-examination of 108 Kato-Katz thick smears (0.9%). In Brazil, the prevalence of *Ascaris* and hookworm (12.2% and 7.3% respectively) was relatively low and a s a result, only 10 and 2 of the 42 re-examined slides were positive for *Ascaris* and hookworm. Following QC, two out of the 42 re-examined slides (4.8%) revealed a discrepancy for *Ascaris* egg counts. No discrepancies were identified for *Trichuris* because this STH was absent in the study area.

At the species level, 1.8% (8/450), 4.2% (19/450) and 2.0% (9/450) of type 1 discrepancies (false positives or negatives) were detected during re-examination for *Ascaris*, *Trichuris* and hookworm respectively. Especially in Ethiopia, a high number of type 1 discrepancies were detected for *Trichuris* (7.9%). Type 2 discrepancies (difference >10 eggs when FECs ≤100 eggs) for *Ascaris*, *Trichuris* or hookworm were detected in 2.0% (9/450), 1.6% (7/450) and 1.1% (5/450) of the re-examined samples, with the highest number of type 2 discrepancies being detected in Tanzania for *Trichuris* (7.1%). Type 3 discrepancies were mostly detected for *Ascaris* (2.9%), while type 3 discrepancies were detected only twice for *Trichuris* (0.4%) (Lao PDR (1/108) and Tanzania (1/84)) and once for hookworm (0.2%) in Lao PDR.

#### Prevalence of any infection and moderate-to-heavy intensity infections following quality control on fecal egg counts

The prevalence and intensity of STH infections at baseline were calculated using the original data (prior to QC on FEC) and the final dataset (containing corrected values following QC of FECs) and compared (**Tables 2 and S1**). All prevalence results were unaffected by possible changes to the data due to QC, except in Ethiopia where prevalence was reduced with 0.2 percentage points for each STH species and in Tanzania, where *Ascaris* prevalence increased by 0.3 percentage points. Changes in the distribution of MHI infections was also minimal (**Table 2**). In Brazil and Lao PDR, implementation of QC on the FEC did not alter the prevalence of MHI infections in any of the STH species. In Ethiopia however, the percentage of MHI infections increased by 0.2 percentage points for *Ascaris* and hookworm and decreased by 0.2 percentage points for *Trichuris* infections. In Tanzania, the number of MHI infections between pre- and post-QC datasets remained unchanged for *Ascaris*, but increased from 67.3% to 67.6% for *Trichuris* and from 1.6% to 3.8% for hookworm infections.

**Table 2:** Prevalence of any infection and moderate-to-heavy intensity infections before and after quality control on fecal egg counts. The prevalence of each soil-transmitted helminth in each study site was calculated based on the duplicate Kato-Katz results at baseline using both the original dataset (Pre-QC FEC dataset) as well as that dataset after adjusting for discrepancies detected during quality control (QC) on the fecal egg counts (Post-QC FEC dataset). The infection intensity in both datasets was classified into light and moderate-to-heavy intensity (MHI) according to the thresholds set by the World Health Organization [10]. Values that differ between both datasets are highlighted in bold font.

		Pre-QC FEC			Post-QC FEC		
Country	STH species	Any intensity (%)	Light (%)	MHI (%)	Any intensity (%)	Light (%)	MHI (%)
**Brazil**	*Ascaris*	12.2	2.8	9.3	12.2	2.8	9.3
**n = 246**	*Trichuris*	0.0	0.0	0.0	0.0	0.0	0.0
	Hookworm	7.3	7.3	0.0	7.3	7.3	0.0
**Ethiopia**	*Ascaris*	**33.3**	**22.0**	**11.3**	**33.1**	**21.6**	**11.5**
**n = 495**	*Trichuris*	**33.5**	32.5	**1.0**	**33.3**	32.5	**0.8**
	Hookworm	**22.2**	**21.8**	**0.4**	**22.0**	**21.4**	**0.6**
**Lao PDR**	*Ascaris*	23.5	14.1	9.4	23.5	14.1	9.4
**n = 469**	*Trichuris*	24.5	23.2	1.3	24.5	23.2	1.3
	Hookworm	58.2	45.2	13.0	58.2	45.2	13.0
**Tanzania**	*Ascaris*	**58.1**	**19.0**	39.1	**58.4**	**19.4**	39.1
**n = 315**	*Trichuris*	98.4	**31.1**	**67.3**	98.4	**30.8**	**67.6**
	Hookworm	37.8	**36.2**	**1.6**	37.8	**34.0**	**3.8**

#### Egg reduction rates and classification of drug efficacy following quality control on fecal egg counts

The ERR was calculated using the original data (prior to QC on FEC) and the final dataset (containing corrected values following QC on FECs) and compared. (**Table 3**). This analysis was performed on the complete dataset from Ethiopia, Lao PDR and Tanzania. The dataset of Brazil was excluded from this analysis because it contained too few positive STH cases to accurately assess drug efficacy. Only the ERRs calculated for *Trichuris* in Ethiopia and Tanzania changed after QC (from 47.7% to 44.9% in Ethiopia and from -18.1% to -18.3% in Tanzania). All other ERRs remained unchanged. There was a 100% agreement in the classification of efficacy of albendazole against STHs between the two datasets [2] (**Table 3**). This means that in nine out of nine evaluations (three STH species in three countries), drug efficacy was categorized into the same class.

**Table 3:** Egg reduction rates and drug efficacy classification before and after quality control on fecal egg counts. Using the dataset from Ethiopia, Lao PDR and Tanzania, the egg reduction rates (ERRs) and 95% Confidence Interval (95% CI) were calculated based on the duplicate Kato-Katz data for each soil-transmitted helminth using both the original dataset (Pre-QC FEC) as well as the dataset after adjusting for discrepancies detected during quality control (QC) on the fecal egg counts (FECs) (Post-QC FEC). The ERRs were classified into satisfactory (green), doubtful (yellow) or reduced (red) according to albendazole efficacy thresholds set by the World Health Organization [2].

		Pre- QC FEC				Post-QC FEC			
Country	STH species	n	Mean FEC at baseline (EPG)	Mean FEC at follow-up (EPG)	ERR % + (95%CI)	n	Mean FEC at baseline (EPG)	Mean FEC at follow-up (EPG)	ERR % + (95%CI)
**Ethiopia**	*Ascaris*	135	666.7	0.5	99.9 (99.8–100)	134	688.4	0.5	99.9 (99.8–100)
	*Trichuris*	139	15.1	7.9	**47.7 (31.3–61.4)**	138	14.7	8.1	**44.9 (31.3–61.4)**
	Hookworm	97	21.8	0.9	95.9 (93.0–98.1)	96	22.1	0.9	95.9 (93.0–98.1)
									
**Lao PDR**	*Ascaris*	104	1,205.6	9.3	99.2 (97.9–99.9)	104	1,205.6	9.3	99.2 (97.9–99.9)
	*Trichuris*	107	30.1	17.9	40.5 (12.5–63.3)	107	30.1	17.9	40.5 (12.5–63.3)
	Hookworm	259	155.7	5.9	96.2 (94.2–97.8)	259	155.7	5.9	96.2 (94.2–97.8)
									
**Tanzania**	*Ascaris*	171	1,649.6	42.7	97.4 (92.8–99.7)	172	1,640.2	42.4	97.4 (92.8–99.7)
	*Trichuris*	288	276.3	326.3	**-18.1 (-38.9–1.0)**	288	277.5	328.3	**-18.3 (-38.8–0.6)**
	Hookworm	107	34.0	4.9	85.6 (77.7–91.6)	107	34.0	4.9	85.6 (77.7–91.6)

### Quality control on the data entry

Across the four trials, a total of 28,980 individual data entries were made (**Table 4**) to digitize the duplicate Kato-Katz data. A total of 201 discrepancies (0.69%) were identified when the entered data from either data entry clerk (DEF person 1 and DEF person 2) was compared with the corrected data.

**Table 4:** The number of discrepant data entries revealed by quality control on data entry. Duplicate Kato-Katz results entered by the two data entry clerks were compared to the corrected dataset and the number of discrepancies were reported.

	Brazil (%)		Ethiopia (%)		Lao PDR (%)		Tanzania (%)		Total (%)	
**Person 1**	6/1,710	(0.35)	10/4,488	(0.22)	51/4,656	(1.10)	38/3,636	(1.05)	105/14,490	(0.73)
**Person 2**	0/1,710	(0.00)	6/4,488	(0.13)	28/4,656	(0.60)	62/3,636	(1.71)	96/14,490	(0.66)
**Total**	6/3,420	(0.18)	16/8,976	(0.18)	79/9,312	(0.85)	100/7,272	(1.38)	201/28,980	(0.69)

**Table 5**shows the prevalence of STH infection of any intensity and MHI infections and the ERRs based on the original data from each data entry clerk and the final data file. Overall, the double data entry affected the prevalence of infections of any intensity by less than 1.1 percentage points for all STHs in all study sites. The effect on the percentage of MHI infections did not exceed 0.2 percentage points except for one comparison in Tanzania. At this study site, we noted a 0.6% difference in prevalence of MHI *Trichuris* infections between the dataset of person 2 and the corrected dataset.

**Table 5:** Prevalence of any infection and moderate-to-heavy intensity infections before and after quality control on data entry. For each country and species of soil-transmitted helminth, the prevalence of any infection and moderate-to-heavy intensity (MHI) infections and the egg reduction rates (ERRs) following single albendazole treatment were calculated. This was done using the raw duplicate Kato-Katz data provided by data entry person 1 (P1), data entry person 2 (P2) and the dataset corrected for possible data entry errors (Corrected). The ERRs were classified into satisfactory (green), doubtful (yellow) or reduced (red) according to albendazole efficacy thresholds set by the World Health Organization [2]. Values that differ from the values obtained by analyzing the corrected database are highlighted in bold.

		Prevalence (%)			MHI prevalence (%)			ERR (%) & WHO classification		
		Pre-data entry QC		Post data-entry QC	Pre-data entry QC		Post data-entry QC	Pre-data entry QC		Post data-entry QC
Country	STH species	P1	P2		P1	P2		P1	P2	
**Brazil**	*Ascaris*	12.2	12.2	12.2	9.3	9.3	9.3	_	_	_
	*Trichuris*	0.0	0.0	0.0	0.0	0.0	0.0	_	_	_
	hookworm	7.3	7.3	7.3	0.0	0.0	0.0	_	_	_
**Ethiopia**	*Ascaris*	33.3	33.3	33.3	**11.5**	**11.5**	11.3	99.9	99.9	99.9
	*Trichuris*	**33.3**	33.5	33.5	**0.8**	**0.8**	1.0	**43.5**	**44.9**	47.7
	hookworm	22.2	**22.4**	22.2	**0.6**	**0.6**	0.4	95.9	**95.8**	95.9
**Lao PDR**	*Ascaris*	**23.2**	**23.2**	23.5	9.4	9.4	9.4	**99.4**	99.2	99.2
	*Trichuris*	**24.1**	**24.1**	24.5	1.3	**1.5**	1.3	**40.2**	**49.0**	40.5
	hookworm	**58.0**	**57.1**	58.2	**12.8**	**12.8**	13.0	96.2	**96.3**	96.2
**Tanzania**	*Ascaris*	**58.7**	**59.0**	58.1	39.1	39.1	39.1	97.4	**97.3**	97.4
	*Trichuris*	98.4	98.4	98.4	67.3	**67.9**	67.3	**-19.4**	**-16.6**	-18.1
	hookworm	**38.1**	**38.1**	37.8	1.6	1.6	1.6	**87.2**	**86.0**	85.6

Performing double data entry had little effect on the ERRs for *Ascaris* (differences of ≤0.2%) and hookworm (≤1.6%). For *Trichuris* however, differences in ERRs calculated from the data entry files provided by both data entry clerks and the corrected data file was ≤8.5%. Nevertheless, despite these discrepancies in ERRs, the final classification of the drug efficacy (as based on WHO reference thresholds (WHO, 2013)) remained unaffected (**Table 5**).

## Discussion

One element of performing QC in STH studies is to ensure that the datasets to be used for statistical analysis are of high quality and integrity. For this, laboratory technicians need to be well-trained to correctly quantify the level of infection present in an individual using whatever diagnostic method available. On top of that, a proper data management system should be put in place to assure that data are reliable, complete and accurate. To our knowledge, the current study is the first to present findings on the effects of performing QC on both FECs and data entry on the study results.

### The selection of samples to re-examine should not be based on a predefined list of random sample numbers

In the current studies, the aim was to re-examine 10% of samples, as suggested by WHO and Speich et al., 2015 [2, 4]. Yet, we noted that in most countries it was difficult to reach this 10%. One important reason for this was likely the time sensitivity of these analysis. Kato-Katz slides needed to be re-examined as soon as possible after the initial reading to prevent clearing of the hookworm eggs, which starts as soon as one hour after preparing the slides [4, 11]. Due to time constraints, it was sometimes not possible to complete the re-examination within the one-hour timeframe. Alternatively, an additional 10% of slides could be labelled with a new ID to allow for a QC on hookworm. However, this is not ideal, as differences between slides might be due to preparing a new slide.

At the start of the studies, a list of pre-defined sample numbers to be re-examined was provided (**S3 Info**) to ensure a 10% re-examination rate and prevent selection bias. In retrospect, providing such list of pre-defined sample IDs is not the best way to select samples for re-examination. Although this enabled the reduction of the sample selection bias, it also limited the flexibility of laboratory staff to select those samples they deemed most interesting for QC. For example, when slide A and slide B of the duplicate Kato-Katz provided notably different egg counts, this would not have prompted QC. Nevertheless, it would have been most useful to have these slides re-examined to verify whether the noted discrepancy was due to the variation in preparing a slide of the same sample or if it was actually the result of a reading error from one of the microscopists. In addition, it happened that certain pre-selected individuals were heavily infected and had thousands of STH eggs in their Kato-Katz thick smears. Re-examining these samples is extremely time-consuming, highly demotivating and has little impact on prevalence or infection intensity results. For these reasons, we are not in favor of using a pre-defined list of sample numbers to be re-examined for QC purposes. Rather, we suggest to leave it up to the laboratory supervisor to select what samples to re-examine. When doing single Kato-Katz, this could be done on a random basis. When duplicate Kato-Katz is performed, discrepancies detected between the FEC results of slide A and slide B (e.g. presence/absence of STH eggs or notable difference in faecal egg counts) could prompt a re-examination.

### More discrepancies in FEC are detected in study sites where multiple infections and MHI infections are more prevalent

We applied the Swiss TPH criteria to evaluate the percentage of re-examinations that resulted in the detection of a discrepancy. Overall 14.4% (65/450) of re-examinations required a third reader to provide the final result. During baseline, more interventions of a third reader were required than during follow-up QC. This was likely due to the fact that many slides were negative during follow-up. Interestingly, when applying WHO recommendations (see **Table 1**), the total number of re-examinations that required a third reading increased to 24.4% (110/450 slides; 27.0% at baseline and 19.5% at follow-up). As previously highlighted by Speich and colleagues [4], these observed differences were due to the more strict WHO recommendations. A comparison of the number of re-examined Kato-Katz slides that would require verification by a third reader according to WHO or Swiss-TPH recommendations is provided in **S1 Table**. Since we only applied the Swiss TPH recommendations in these trials, the impact of performing QC according to WHO recommendations on study outcomes could not be assessed and compared.

Notable variability could be seen in the number and type of discrepancies detected across the different countries and STHs. As could be anticipated, a higher number of discrepancies were found in study sites where multiple STH species and MHI infections were more prevalent as it is indeed more difficult to keep focus and easier to make counting mistakes when many eggs are present in the sample and when the sample contains multiple different egg types.

### QC on FECs had a limited impact on the prevalence of any infection and MHI infections, ERRs and the classification of drug efficacy

Interestingly, we found that, independent of the country, the QC that was performed on a subset of samples had little to no impact on important programmatic parameters like STH prevalence of any infection, the prevalence of MHI infections and ERRs. There was a perfect agreement between the original and corrected datasets in classifying drug efficacy for each STH species (9 out of 9 classifications were identical). In this context, it is however important to mention an essential limitation of this study. Prior to the start of these studies, microscopists were informed that QC would be performed on a subset of the samples. This might have affected their level of focus and attention during the evaluations, resulting in data that was already of relatively good quality. The quality of the pre-QC dataset may have been lower if microscopists were not aware that their work would be submitted to QC. Ideally, a comparison should have to be made between data obtained from performing QC on samples of a study where microscopists were not aware that QC was going to be performed.

### Double data entry reduces the number of erroneous data entries but has a limited impact on the prevalence of any infection and MHI infections, ERRs and the classification of drug efficacy

One of the most important steps in the process of processing data from epidemiological studies or clinical trials is data entry from physical data forms into a digital data sheet using a computer or any other data entry system. Whenever manual data entry is required, the possibility exists of including errors into the transcribed dataset. Depending on the error and the size of the dataset, these errors can significantly affect the results of a study [6]. To prevent the inclusion of such errors during the data entry process, double data entry is recommended since it is far superior than alternative methods (e.g. single entry, reading aloud or visual checking) in reducing the number of errors [5, 6]. It remains superior, regardless of the skill level of a data entry clerk and regardless of whether data is entered twice by one person or once by two separate individuals [5].

Data entry errors were identified in each of our four studies. The presence of a high number of positive individuals and multiple STH species infections increased the number of non-zero entries to be made, which in turn increased the probability of errors. In Brazil for example, where only few *Ascaris* and hookworm infections were diagnosed, the lowest number of data entry errors were noted, whereas in Tanzania, where many children carried multiple STH infections and where infections were often of MHI, we noted a higher number of data entry errors. Differences in the Microsoft Excel skill-level among data entry clerks likely also affected the amount of errors made.

In most cases, performing QC on the data entry had limited impact (<1%) on measurements of prevalence of any infection, MHI infections or ERRs. However, in the case of *Trichuris* in Tanzania, the introduced errors resulted in an 8.5% difference in ERR. While the interpretation of the drug efficacy according to WHO standards did not change, this would mean a considerable difference when the performance of a novel treatment would be evaluated.

Given the total number of discrepancies detected and the little time and resources needed to perform this QC step, we would highly recommend to perform double data entry when digitizing raw data collected on paper. It has been suggested in a number of studies that electronic data collection reduces the number of errors [12, 13], is more time- and cost-efficient [12–15] and yields data that is immediately available for analysis [12, 16, 17]. This could indeed simplify and standardize the reporting and sharing of study results, and hence be of particular interest to country program managers and other stakeholders. Nevertheless, there are still a number of reservations which may hamper quick adoption of electronic data capture such as the need for well-maintained and secure digital repositories, ensuring correct human operation through sufficient training and issues that could disrupt the data processing [17].

### QC should extend beyond checking FECs and data entry

Although performing QC on the FECs and data entry can highlight some discrepancies, there are a number of other steps in the diagnostic process and data management where errors can be introduced and which might be more impactful on overall study results. Most errors, may actually be introduced at the source, during the collection and preparation of the diagnostic sample (e.g. improper storage conditions of collected stool samples, waiting too long to process samples, not homogenizing stool samples prior to taking an aliquot for diagnosis, contamination of tools and bench top environment used to prepare samples, misuse of materials or applying suboptimal methodology). An inclusive training of the personnel and strict adherence to study SOPs is thus of paramount importance for the production of high-quality study data.

In the current studies, staff were familiarized with study documents and SOPs prior to the start of the trials. Technicians were trained on how to perform the diagnostic method and on how to read Kato-Katz slides using a couple of ‘dummy’ stool samples. Yet, it would be of value to adopt a more comprehensive training where slide readers are requested to identify and count the number of STH eggs in a predesigned set of Kato-Katz thick smears or digital images thereof, to independently evaluate reader competence levels. Recently, a similar QC system was piloted for the molecular detection of STHs, *Strongyloides stercoralis* and *Schistosoma* spp. [18].

## Conclusions

In this study, we showed that re-examining a subset of Kato-Katz thick smears and performing double data entry leads to the identification of discrepancies and finally results in a dataset of improved quality. But, despite these improvements, the overall study outcomes were not significantly affected. This however does not mean that the implementation of QC is redundant. Performing FECs and digitizing the results are only two steps of a more elaborate diagnostic process, of which certain steps cannot be subjected to QC (e.g. certain steps in the sample collection and preparation process). Therefore, in order to ensure the higher possible level of data quality, it is still recommended to continue performing QC wherever possible while providing proper training to study personnel and stimulating adherence to SOPs.

## Supporting information

S1 Info Standard operating procedure on how to perform duplicate Kato-Katz thick smears. (PDF)

S2 Info The record forms used to register duplicate Kato-Katz results and the results of Quality control. (PDF)

S3 Info Standard Operating Procedure on how to perform quality control including the list of pre-defined Subject IDs. (PDF)

S4 Info Data entry form (Excel). (XLSX)

S5 Info Standard operating procedure on how to compare two data entry files using the Excel “inquire” add-on. (PDF)

S6 Info Raw data used for analysis of quality control on the fecal egg counts. (XLSX)

S1 Table Prevalence of any infection and moderate-to-heavy intensity infections before and after quality control on fecal egg counts including 95% confidence interval. (DOCX)

S2 Table The number of re-examined Kato-Katz slides that would require verification by a third reader according to WHO or Swiss-TPH recommendations at baseline (BL) or during follow-up (FU) screening of the study participants. (DOCX)
