# Supplementary material for: An in-depth report of quality control on Kato-Katz and data entry in four clinical trials evaluating the efficacy of albendazole against soil-transmitted helminth infections
Source: PLoS Negl Trop Dis. 2020 Sep 21;14(9):e0008625. doi: 10.1371/journal.pntd.0008625 (PMC7549791; doi:10.1371/journal.pntd.0008625)
Supplement: S2 Info — (PDF) [file pntd.0008625.s002.pdf]

RF 02: Kato-Katz Examination

Study: OPP1120972

Date of examination (DD/MMM/YYYY): __ / ___ / 2 0 1 7

☐ Baseline ☐ Follow up

Subject ID (LAXXX)	Slide A/B	Number of *Ascaris* eggs (XXXX)	Number of *Trichuris* eggs (XXX)	Number of Hookworm eggs (XXX)	Time to read slide (min:sec)	Initials of the examiner (XXX)	Selected for stool preservation? *
B R	A				:		☐ Yes ☐ No
	B				:		
B R	A				:		☐ Yes ☐ No
	B				:		
B R	A				:		☐ Yes ☐ No
	B				:		
B R	A				:		☐ Yes ☐ No
	B				:		
B R	A				:		☐ Yes ☐ No
	B				:		

* During baseline examination, only stool samples found to contain at least 13 eggs on duplicate Kato-Katz or at least 15 eggs on Mini-FLOTAC for at least one of the three STH need to be preserved. During follow-up examination, all samples need to be conserved (See SOP 11).

Signature investigator: ____________________ **Date (DD/MMM/YYYY)** __ / ___ / 2 0 1 7

RF 03: Kato Katz Quality Control

Study: OPP1120972

Date of Quality Control (DD/MMM/YYYY): __ __ / __ __ __ / 2 0 1 7

Subject ID (XXXXX)	Slide	Number of *Ascaris* eggs (XXXX)			Number of *Trichuris* eggs (XXX)			Number of Hookworm eggs (XXX)			Initials of 2nd examiner (XXX)
		Quality control exam.	First exam.	Meeting quality criteria*	Quality control exam.	First exam.	Meeting quality criteria*	Quality control exam.	First exam.	Meeting quality criteria*	
B R __ __ __	A			☐ Yes ☐ No			☐ Yes ☐ No			☐ Yes ☐ No	
	B			☐ Yes ☐ No			☐ Yes ☐ No			☐ Yes ☐ No	
B R __ __ __	A			☐ Yes ☐ No			☐ Yes ☐ No			☐ Yes ☐ No	
	B			☐ Yes ☐ No			☐ Yes ☐ No			☐ Yes ☐ No	

Comments:

*No false negatives/positives; difference in egg counts: ≤10 eggs difference when the total number of eggs counted ≤100 eggs (not eggs per gram of stool, but number of eggs counted under the microscope) or ≤ 20% when more than 100 eggs are counted. If sample does not meet these quality criteria, please follow the protocol described in SOP 10.

Signature investigator : ______________________ **Date (DD/MMM/YYYY) :** __ __ / __ __ __ / 2 0 1 7
