# Supplementary material for: An in-depth report of quality control on Kato-Katz and data entry in four clinical trials evaluating the efficacy of albendazole against soil-transmitted helminth infections
Source: PLoS Negl Trop Dis. 2020 Sep 21;14(9):e0008625. doi: 10.1371/journal.pntd.0008625 (PMC7549791; doi:10.1371/journal.pntd.0008625)
Supplement: S1 Table — (DOCX) [file pntd.0008625.s007.docx]

**Supplementary Table 1: Prevalence of any infection and moderate-to-heavy intensity infections before and after quality control on fecal egg counts including 95% confidence intervals.** The prevalence of each soil-transmitted helminth in each study site was calculated based on the duplicate Kato-Katz results at baseline using both the original dataset (Pre-QC FEC dataset) as well as that dataset after adjusting for discrepancies detected during quality control (QC) on the fecal egg counts (Post-QC FEC dataset). The infection intensity in both datasets was classified into light and moderate-to-heavy intensity (MHI) according to the thresholds set by the World Health Organization [[10](#_ENREF_10)]. Values that differ between both datasets are highlighted in bold font.

|  |  | **Pre-QC FEC** | | |  | **Post-QC FEC** | | |
| --- | --- | --- | --- | --- | --- | --- | --- | --- |
| **Country** | **STH species** | Any intensity (% + 95%CI) | Light  (% + 95%CI) | MHI  (% + 95%CI) |  | Any intensity  (% + 95%CI) | Light  (% + 95%CI) | MHI  (% + 95%CI) |
| **Brazil** | *Ascaris* | 12.2 (8.1-16.3) | 2.8 (0.7-4.9) | 9.3 (5.7-12.9) |  | 12.2 (8.1-16.3) | 2.8(0.7-4.9) | 9.3 (5.7-12.9) |
| **n = 246** | *Trichuris* | 0.0 (0.0-0.0) | 0.0 (0.0-0.0) | 0.0 (0.0-0.0) |  | 0.0 (0.0-0.0) | 0.0 (0.0-0.0) | 0.0 (0.0-0.0) |
|  | Hookworm | 7.3 (4.0-10.6) | 7.3 (4.0-10.6) | 0.0 (0.0-0.0) |  | 7.3 (4.0-10.6) | 7.3 (4.0-10.6) | 0.0 (0.0-0.0) |
|  |  |  |  |  |  |  |  |  |
| **Ethiopia** | *Ascaris* | **33.3 (29.1-37.5)** | **22.0 (18.4-25.6)** | **11.3 (8.5-14.1)** |  | **33.1 (29.0-37.2)** | **21.6 (18.0-25.2)** | **11.5 (8.7-14.3)** |
| **n = 495** | *Trichuris* | **33.5 (29.3-37.7)** | 32.5 (28.4-36.6) | **1.0 (0.1-1.9)** |  | **33.3 (29.1-37.5)** | 32.5 (28.4-36.6) | **0.8 (0.0-1.6)** |
|  | Hookworm | **22.2 (18.5-25.9)** | **21.8 (18.2-25.4)** | **0.4 (0.0-1.0)** |  | **22.0 (18.4-25.6)** | **21.4 (17.8-25.0)** | **0.6 (0.0-1.3)** |
|  |  |  |  |  |  |  |  |  |
| **Lao PDR** | *Ascaris* | 23.5 (19.7-27.3) | 14.1 (11.0 17.2) | 9.4 (6.8-12.0) |  | 23.5 (19.7-27.3) | 14.1 (11.0 17.2) | 9.4 (6.8-12.0) |
| **n = 469** | *Trichuris* | 24.5 (20.6-28.4) | 23.2 (19.4-27.0) | 1.3 (0.3-2.3) |  | 24.5 (20.6-28.4) | 23.2 (19.4-27.0) | 1.3 (0.3-2.3) |
|  | Hookworm | 58.2 (53.7-62.7) | 45.2 (40.7-49.7) | 13.0 (10.0-16.0) |  | 58.2 (53.7-62.7) | 45.2 (40.7-49.7) | 13.0 (10.0-16.0) |
|  |  |  |  |  |  |  |  |  |
| **Tanzania** | *Ascaris* | **58.1 (52.7-63.5)** | **19.0 (14.7-23.3)** | 39.1 (33.7-44.5) |  | **58.4 (53.0-63.8)** | **19.4 (15.0-23.8)** | 39.1 (33.7-44.5) |
| **n = 315** | *Trichuris* | 98.4 (97.0-99.8) | **31.1 (26.0-36.2)** | **67.3 (62.1-72.5)** |  | 98.4 (97.0-99.8) | **30.8 (25.7-35.9)** | **67.6 (62.4 -72.8)** |
|  | Hookworm | 37.8 (32.4-43.2) | **36.2 (30.9-41.5)** | **1.6 (0.2-3.0)** |  | 37.8 (32.4-43.2) | **34.0 (28.8-39.2)** | **3.8 (1.7-5.9)** |
