# Supplementary material for: An in-depth report of quality control on Kato-Katz and data entry in four clinical trials evaluating the efficacy of albendazole against soil-transmitted helminth infections
Source: PLoS Negl Trop Dis. 2020 Sep 21;14(9):e0008625. doi: 10.1371/journal.pntd.0008625 (PMC7549791; doi:10.1371/journal.pntd.0008625)
Supplement: S2 Table — (DOCX) [file pntd.0008625.s008.docx]

**Supplementary Table 2: The number of re-examined Kato-Katz slides that require verification by a third reader according to WHO or Swiss-TPH recommendations at baseline (BL) or during follow-up (FU) screening of the study participants.**

|  |  | **WHO** | | **Swiss TPH** | |
| --- | --- | --- | --- | --- | --- |
|  |  | n | % | n | % |
| **Brazil** | BL | 7/42 | 16.7% | 2/42 | 4.8% |
|  | FU | NA | NA | NA | NA |
| **Ethiopia** | BL | 47/146 | 32.2% | 33/146 | 22.6% |
|  | FU | 10/70 | 17.1% | 10/70 | 14.3% |
| **Lao PDR** | BL | 8/64 | 12.5% | 3/64 | 4.7% |
|  | FU | 1/44 | 2.3% | 0/44 | 0.0% |
| **Tanzania** | BL | 18/44 | 40.9% | 10/44 | 22.7% |
|  | FU | 17/40 | 42.5% | 7/40 | 17.5% |
| **Total** | BL | 80/296 | 27.0% | 48/296 | 16.2% |
|  | FU | 30/154 | 19.5% | 17/154 | 11.0% |
| **Total** | ALL | 110/450 | 24.4% | 65/450 | 14.4% |
